# Differentiation of Morphological Traits and Genome-Wide Expression Patterns between Rice Subspecies *Indica* and *Japonica*

**DOI:** 10.3390/genes14101971

**Published:** 2023-10-21

**Authors:** Meixia Wang, Lei Huang, Yixuan Kou, Danqi Li, Wan Hu, Dengmei Fan, Shanmei Cheng, Yi Yang, Zhiyong Zhang

**Affiliations:** 1 Laboratory of Subtropical Biodiversity, Jiangxi Agricultural University, Nanchang 330045, China; yixuankou@163.com (Y.K.); lidq@lsbg.cn (D.L.); hwan603@163.com (W.H.); dmf.625@163.com (D.F.); shan13110700028@163.com (S.C.); yangyi9201@sina.com (Y.Y.); 2 College of Life Sciences, Shaanxi Normal University, Xi’an 710119, China; caspar@snnu.edu.cn; 3 Key Laboratory of Ecology of Rare and Endangered Species and Environmental Protection (Guangxi Normal University), Ministry of Education, Guilin 541004, China; 4 Lushan Botanical Garden, Jiangxi Province and Chinese Academy of Sciences, Jiujiang 332900, China

**Keywords:** domestication, rice, genome-wide expression patterns, morphological traits

## Abstract

Changes in gene expression patterns can lead to the variation of morphological traits. This phenomenon is particularly evident in recent evolution events such as crop domestication and responses to environmental stress, where alterations in expression levels can efficiently give rise to domesticated syndromes and adaptive phenotypes. Rice (*Oryza sativa* L.), one of the world’s most crucial cereal crops, comprises two morphologically distinct subspecies, *Indica* and *Japonica*. To investigate the morphological divergence between these two rice subspecies, this study planted a total of 315 landrace individuals of both *Indica* and *Japonica* under identical cultivation conditions. Out of the 16 quantitative traits measured in this study, 12 exhibited significant differences between the subspecies. To determine the genetic divergence between *Indica* and *Japonica* at the whole-genome sequence level, we constructed a phylogenetic tree using a resequencing dataset encompassing 95 rice landrace accessions. The samples formed two major groups that neatly corresponded to the two subspecies, *Indica* and *Japonica*. Furthermore, neighbor-joining (NJ) trees based on the expression quantity of effectively expressed genes (EEGs) across five different tissues categorized 12 representative samples into two major clades aligning with the two subspecies. These results imply that divergence in genome-wide expression levels undergoes stabilizing selection under non-stressful conditions, with evolutionary trends in expression levels mirroring sequence variation levels. This study further supports the pivotal role of changes in genome-wide expression regulation in the divergence of the two rice subspecies, *Indica* and *Japonica*.

## 1. Introduction

Early in 1970s, Wilson et al. proposed that divergence in gene expression regulation plays a significant role in evolution [1,2]. This hypothesis was subsequently supported by a series of studies in various animals and plants, including genes related to domestication in cultivated plants and genes leading to morphological variation among varieties [3]. For example, mutations in the upstream regulatory region of the *tb1* gene in maize altered the branching pattern of individuals [4]; regulatory variation in the *qSH1* gene in rice led to changes in seed shattering [5,6]; expression regulatory variation in the *fw2.2* gene in tomatoes resulted in changes in fruit size [3]; alterations in the expression of the *Q* gene in wheat affected its inflorescence structure, and so on [3]. Purugganan and Fuller (2009) summarized that of the identified nine domestication genes, eight encoded transcriptional activators, suggesting that the domestication process may involve not only variations in the gene regions encoding amino acids but also variations in gene regulatory regions [7]. Doebley et al. (2006) postulated that the proportion of genes with transcriptional regulatory roles might be higher in determining morphological differences between crops and their ancestral species than genes involved in amino acid substitution [3].

Compared to sequence variation in the genome, variation at the transcriptome level could change with time and space. This form of expression regulatory variation is more closely associated with phenotypic variation and is most likely to be preserved through natural and artificial selection [1,8]. Especially when plants face sudden environmental stress, changes in expression levels can respond to environmental changes quickly, providing hope for the survival of plants. For example, Groen et al. (2020) found that under drought conditions, the selection intensity calculated based on transcriptome data was stronger than that in the control group under moist conditions [9]. Due to the variability at the transcriptome level, studies are often influenced by a series of factors, such as the external environment, the choice of tissue type, etc. Therefore, the role of expression variation in population divergence and adaptive evolution still requires further research.

**Figure 1:**
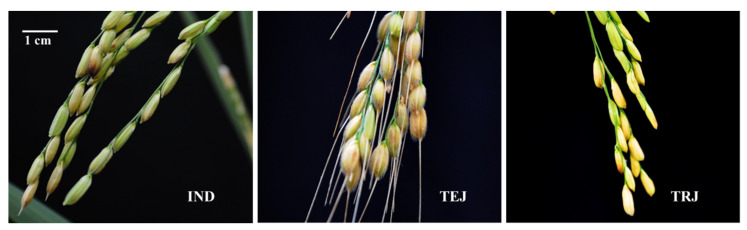
The apex of panicle in *Indica* and *Japonica* (TEJ and TRJ) rice.

Rice (*O. sativa* L.) is one of the world’s most important crops, with two subspecies, *Indica* and *Japonica*, characterized by differences in morphology, habitats/ecosystems, geographic distribution, cultivation culture, etc. (Figure 1) [10,11]. Furthermore, *Indica* rice mainly comprises the IND group, while *Japonica* rice predominantly encompasses *temperate japonica* (TEJ) and *tropical japonica* (TRJ) [12]. *Indica* rice is primarily grown in low-latitude regions such as the tropics and subtropics, while *Japonica* rice is cultivated in high-latitude areas with relatively lower temperatures and high altitudes [13,14].

Despite a series of studies on the phenotypic and whole-genome sequence divergence between the two subspecies of rice [11,12,13,14], we still cannot clearly understand whether the phenotypic separation between the two subspecies of rice is consistent with the whole-genome sequence divergence, especially regarding expression differentiation. Therefore, this study needs to address the following two questions: (1) What are the morphological differences between the two subspecies of rice? (2) Is the expression variation at the whole genome level consistent with the trend of DNA sequence divergence between the two subspecies of rice?

## 2. Materials and Methods

### 2.1. Sample Collection for Phenotypic Observation and RNA-Seq

To explore the divergence between *Indica* and *Japonica* rice, we chose 21 accessions covering two subspecies of rice for phenotypic measurements (Table 1). These accessions are all landraces, including 13 *Japonica* (9 TEJ and 4 TRJ) and 8 *Indica* (IND) accessions. Each accession consisted of 15 plants, totaling 315 plants. We cultivated these accessions in the phytotron under controlled conditions with a temperature of 30 °C (day, 8:00–20:00) and 25 °C (night) and a humidity level of 50%.

We referred to “Descriptors for wild and cultivated rice” (Bioversity International, IRRI and Africa Rice Center, 2007) and selected 16 quantitative traits for measurement (Table 2) [15]. We calculated the coefficient of variation (CV) and performed one-way analysis of variance (ANOVA). Due to significant phenotypic divergence between *temperate japonica* (TEJ) and *tropical japonica* (TRJ) within *Japonica*, we conducted separate analyses for these two groups.

The accessions for RNA sequencing (RNA-seq) were a subset of those for phenotypic observation, including 12 accessions (6 *Indica* and 6 *Japonica*) from the above 21 samples for studying phenotype (Table 1). The RNA-seq dataset was from our previously published research [16]. Each accession was cultivated with a minimum of five individuals to obtain RNAs at various developmental stages. We collected five types of tissues, i.e., leaves at the seedling stage (S), flag leaves (LH) and panicles (PH) at the heading stage, as well as flag leaves (LM) and panicles (PM) at the milk stage [16], for RNA sequencing using the Illumina HiSeq 2000 platform.

### 2.2. Whole-Genome Resequencing Data

To explore the divergence between *Indica* and *Japonica* at the whole-genome sequence level, we generated a resequencing dataset comprising 95 rice landraces, consisting of 56 accessions from the *Indica* subspecies and 39 accessions from the *Japonica* subspecies (Table S1). Out of these, the resequencing data for 82 accessions were downloaded from the 3000 Rice Genome Project (3K RGP) [17] and the remaining 13 accessions were from our previously published research [16]. These 95 accessions cover all accessions for RNA-seq and some accessions for phenotype observation.

### 2.3. Phylogeny Based on SNPs from Resequencing Data

We acquired an average of 14G of clean resequencing data for each accession. We first mapped the short clean reads to the *O. sativa* Nipponbare genome (MSU version 7.0) using the BWA-MEM algorithm. On average, we observed that 98.5% of the reads were successfully aligned to the reference genome, with a mean sequencing depth of 16.6× and an average coverage of 93.1% across the reference genome [18]. We utilized the HaplotypeCaller in the Genome Analysis Toolkit (GATK 3.5) for SNP calling, with parameters of “-stand_emit_conf 10, -stand_call_conf 30”. To minimize false positives, we filtered the raw SNPs through variant quality score recalibration (VQSR), resulting in a total of 9,282,239 reliable SNPs for further analyses.

We employed a sliding window approach with a 100 kb window, sliding in 10 kb steps, to scan the whole genome and estimate the nucleotide diversity (π) of the two subspecies, respectively. We also calculated the genetic divergence (*F*_ST_) between *Indica* and *Japonica* using non-overlapping 10 kb windows, sliding in 10 kb steps, by vcftools [19]. A principal component analysis (PCA) was performed to assess the relatedness and clustering among all samples, utilizing the GCTA software (version 1.24.4) [20]. Furthermore, we computed pairwise genetic distances for all 95 samples, generating a genetic distance matrix using our in-house PERL scripts. Following this, we constructed a neighbor-joining (NJ) tree using MEGA (version 6.0) based on this distance matrix [21].

### 2.4. Phylogeny Based on RNA-Seq Data

Following the removal of low-quality reads, we successfully acquired over 30 million clean read pairs from each RNA-seq sample, with an average clean data size of approximately 6G per sample. Subsequently, these clean reads were aligned to the reference genome (*O. sativa* Nipponbare, MSU version 7.0) using TopHat2 [22], achieving mapping rates that ranged from 89.7% to 92.1%, varying depending on the tissues. To quantify expression levels, we employed the HTSeq.scripts.count feature in the software of HTSeq (version 0.6.1p1) [23]. Only reads that uniquely mapped to the reference genome were selected for gene expression calculations. We normalized gene expression using edgeR [24], computing count-per-million (cpm) and reads per million reads (RPKM). During normalization, we applied the TMM (trimmed mean of M-values) method. We defined a gene as effectively expressed (EEG) if it had at least one mapped read count per million in more than one of the samples (rowSums(cpm(d) > 1) ≥ 1). In total, we identified 29,818 EEGs, which represent genes that were effectively expressed in at least one tissue.

To investigate the evolutionary relationships among the samples, we computed pairwise distances between all 12 samples based on the expression levels of EEGs, utilizing our custom R scripts. This analysis generated a genetic distance matrix for each tissue. Subsequently, we utilized the distance matrixes to construct neighbor-joining (NJ) trees employing PHYLIP (version 3.696) [25].

### 2.5. Identifying Differentially Expressed Genes (DEGs) between Indica and Japonica Rice

We identified differentially expressed genes (DEGs) between *Indica* and *Japonica* rice in each tissue using edgeR [24]. Our criteria for detecting DEGs included a significance threshold with FDR (adjusted *p*) < 0.05 and |log_2_^FC^| > 2. Additionally, we performed gene ontology (GO) enrichment analysis for the DEGs in each tissue. We selected significant GO terms in the biological process (BP) category based on a Fisher’s exact test with a *p* value < 0.05.

## 3. Results

### 3.1. Morphological Variation between Two Subspecies of Rice

To quantify the morphological distinctions between *Indica* and *Japonica* rice, we performed a diversity assessment and differential analysis of 16 quantitative traits across 138 plants. As shown in Table 2, the coefficient of variation (CV) for three traits, namely, the attitude of flowering panicle branches, attitude of seed setting panicle branches, and awn length, was greater than 1. One-way analysis of variance (ANOVA) revealed that, except for the attitude of flowering panicle branches, attitude of seed setting panicle branches, anther length, and number of panicles per plant, the other 12 traits showed significant differences between the subspecies (*p* < 0.05).

**Figure 2:**
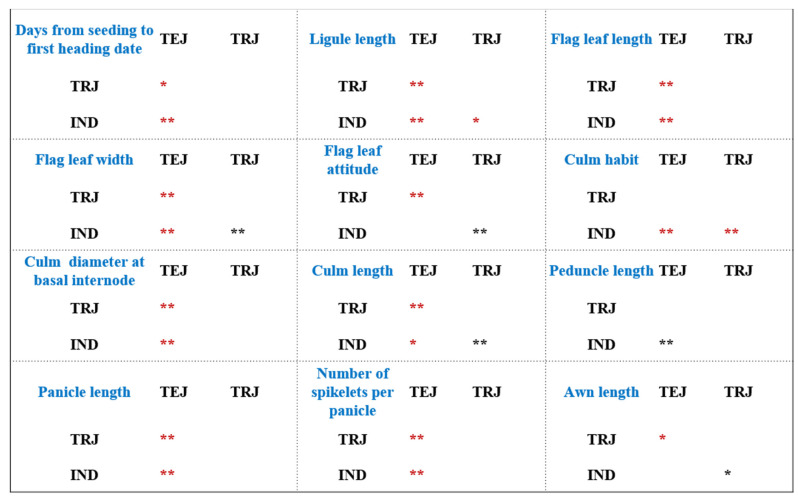
LSD pairwise comparisons among rice groups of 12 quantitative traits that exhibited significant divergence between subspecies. * means *p*-values < 0.05, ** means *p*-values < 0.01; the * in red indicates that the measured value of a trait in the horizontal population is higher than that in the vertical population.

As observed from Table 2 and Figure 2, in terms of nutrition-related traits, the ligule length of the *Indica* subspecies (IND) was significantly longer than that of the *Japonica* subspecies (TEJ and TRJ). IND exhibited significantly greater flag leaf length and width than TEJ. Whereas there was no significant difference in flag leaf attitude between IND and TEJ, it was notably larger in TRJ. Furthermore, based on culm habit, we found that the *Japonica* subspecies (TEJ and TRJ) exhibited significantly denser plant growth compared to the *Indica* subspecies. However, IND was thicker than TEJ in terms of culm diameter at basal internode, and IND was significantly taller than TEJ but shorter than TRJ in terms of culm length (Table 2, Figure 2). In terms of reproductive growth-related traits, IND flowered later than TEJ under our cultivate conditions (12 h of daylight per day). The peduncle length of IND was shorter than that of TEJ, but IND had significantly longer panicles and higher number of spikelets per panicle than TEJ. Additionally, the awn length of IND was significantly shorter than that of TRJ (Table 2, Figure 2).

### 3.2. Divergence at Genome-Wide DNA Sequence Level and Expression Level between the Two Subspecies of Rice

**Figure 3:**
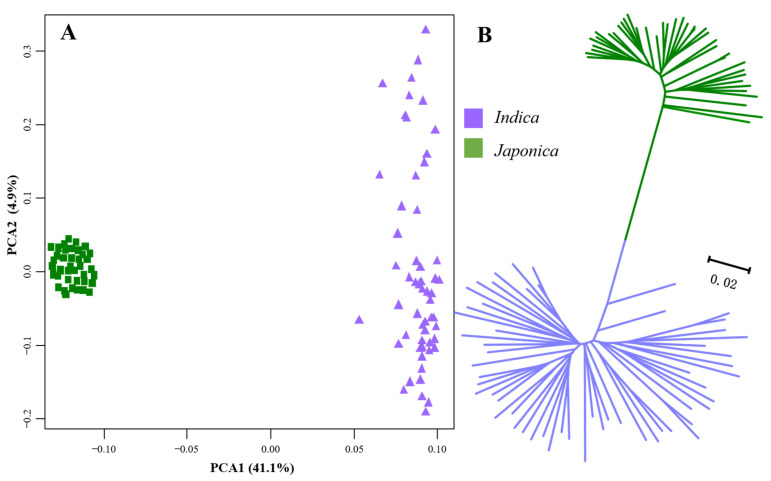
Analyses of population genetic divergence of all 95 rice accessions based on the total SNPs of the resequencing data. (**A**) Principal components analysis (PCA). (**B**) Neighbor-joining (NJ) tree.

Our initial investigation focused on the nucleotide diversity and population genetic divergence among all samples based on the SNPs extracted from the resequencing data. As anticipated, we observed a higher level of nucleotide diversity within the *Indica* subspecies (π = 0.0031) compared to the *Japonica* subspecies (π = 0.0018). The genetic divergence (*F*_ST_) between these two subspecies was 0.5569. In our principal component analysis (PCA), PCA1 accounted for a substantial 41.1% of the variations, effectively grouping all samples into two distinct clusters corresponding to the *Indica* subspecies and the *Japonica* subspecies, with no overlap (Figure 3A). Similarly, the neighbor-joining (NJ) tree clearly revealed that all 95 samples segregated into two major groups, aligning with the two subspecies, i.e., *Indica* and *Japonica* rice (Figure 3B).

**Figure 4:**
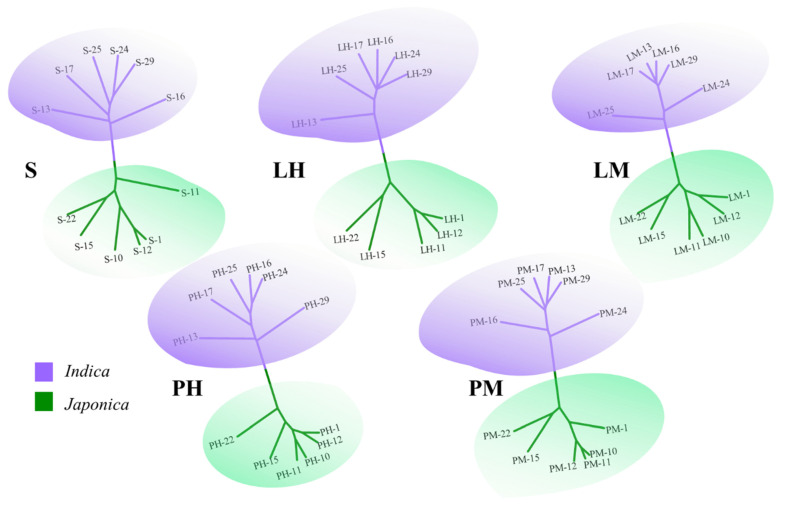
NJ trees based on the expression quantity of the effectively expressed genes in five tissues: leaves at the seedling stage (S), flag leaves (LH) and panicles at the heading stage (PH), flag leaves (LM) and panicles at the milk stage (PM).

In our further analysis on the RNA-seq data, we identified a consistent pattern with the results based on the resequencing data. Specifically, the NJ tree constructed based on the expression levels of effectively expressed genes (EEGs) in each of the five tissues distinctly clustered all samples into two major clades, corresponding to *Indica* and *Japonica* rice (Figure 4).

Furthermore, the DEGs between *Indica* and *Japonica* rice were identified across five tissues. There were an average of 3000 DEGs (2498 to 3613) between the two subspecies, accounting for 12.4% of the number of EEGs on average (Table 3). Moreover, the numbers of up-regulated DEGs of *Japonica* rice to *Indica* rice were larger than that of down-regulated DEGs (Table 3). We further conducted functional enrichment analysis of these DEGs, respectively, and selected significantly enriched GO terms. For example, there were 16 significantly enriched GO terms in PH and most of them were related to pollination and reproduction, indicating the significant expression differentiation of reproductive-related genes in the panicles of the two rice subspecies at the heading stage (Figure S1).

## 4. Discussion

### 4.1. Significant Divergence in Morphological Traits between the Two Subspecies of Rice

Morphological variation serves as the foundation for adaptive evolution and natural selection [26]. Crops domestication also began with the selection of phenotypes. Apart from the emergence of domestication syndromes such as loss of shattering and enlargement of fruits, landrace populations from different regions have also developed specific phenotypic characteristics to adapt to their local environments. These morphological traits encompass both qualitative traits determined by single genes or major-effect genes and quantitative traits determined by polygenic effects [3,27]. For example, some rice varieties can flower earlier and expand their cultivation range to higher latitudes through changes in minor-effect gene *DTH2* [28].

In this study, we observed a total of 16 quantitative traits, with 12 of them showing significant divergence among subspecies. In general, the ligule length of the *Indica* subspecies (IND) was significantly longer than that of the *Japonica* subspecies (TEJ and TRJ), and *Japonica* subspecies (TEJ and TRJ) plants exhibited significantly denser growth (culm habit). Therefore, the ligule length and culm habit could be used as distinguishing characteristics between these two subspecies.

Previous studies commonly classified IND and TEJ as the *Indica* and *Japonica* subspecies, which we refer to as subspecies in the narrow concept [12]. Our research found that IND had significantly longer and wider flag leaves, a thicker culm, a greater culm height, a later heading date, longer panicles, and more spikelets per panicle compared to TEJ, but IND had a shorter peduncle length than TEJ (Figure 2). These results also partially explain why the *Indica* subspecies has higher yields than the *Japonica* subspecies [29].

It is worth noting that TRJ significantly exceeded TEJ and IND in terms of flag leaf width and attitude, culm length, and awn length (Figure 2). These four traits not only help us distinguish between them morphologically but also guide us to further investigate why TRJ is more suitable for growing in higher temperature environments than TEJ [12,30]. In addition, TRJ and IND did not show significant differences in traits such as days from seeding to the first heading date, flag leaf length, culm diameter at the basal internode, panicle length, or number of spikelets per panicle, but they were significantly different from TEJ in these traits (Figure 2). This may be related to both TRJ and IND adapting to higher temperature environments [12,30].

### 4.2. Divergence between Indica and Japonica Rice at Both DNA Sequence and Expression Levels

It is well known that divergence across the entire genome among species or populations exists at both sequence and expression levels. This variation depends on the species itself and various factors behind the divergence [31,32]. Previous studies have shown that expression variation plays a crucial role in adaptive evolution and speciation processes [33,34]. For instance, King and Wilson (1975) proposed that the significant differences between humans and chimpanzees is not caused by the changes in protein-coding genes but by the alterations in gene expression regulatory mechanisms [1]. In a speciation study of two crow species (*Corvus* [*corone*] *corone* and *Corvus* [*corone*] *cornix*), there was almost no difference in 25 intron sequences examined between them, but significant differences were observed at the expression level, indicating the significant effects of expression variation on species divergence [34]. Furthermore, Martinez-Fernandez et al. (2010) found that the rate of divergence between two snail ecotypes at the sequence, expression, and protein levels was 3%, 4%, and 7%, respectively, suggesting that the degree of species divergence is least obvious at the sequence level, most pronounced at the phenotypic level, and intermediate at the expression regulation level [35]. Therefore, changes in gene expression patterns can lead to the variation of morphological traits during evolution.

**Figure 5:**
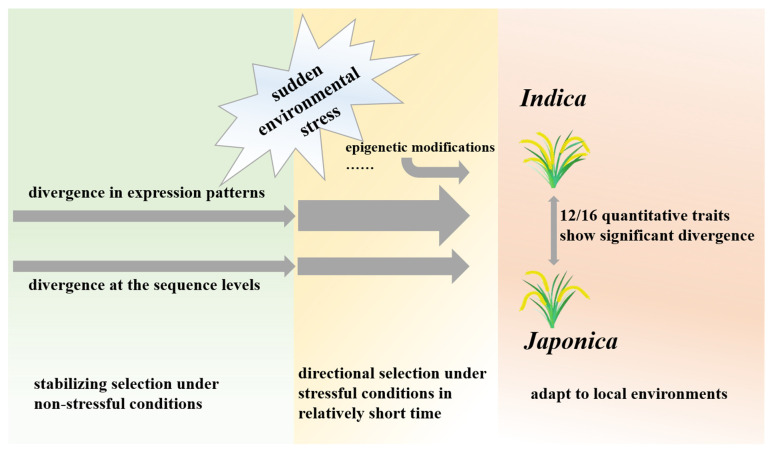
The variations at both DNA sequence and expression levels behind the phenotypic divergence of the two rice subspecies.

In this study (Figure 5), the phylogenetic trees based on the whole-genome sequences and the gene expression in different tissues were consistent in topologies. This indicates that the divergence in genome expression is subjected to stabilizing selection under non-stressful conditions, and the evolution trends in expression variation levels are consistent with sequence variation levels [9]. However, when populations are subjected to environmental stress, epigenetic modifications (e.g., DNA methylation) and gene expression can be changed, leading to faster adaptation to new growth environments compared to sequence mutations [36,37,38,39]. Changes in the expression of one gene can affect other related genes, so genome-wide expression regulation might evolve more rapidly than nucleotide variation [34,39]. By far the most domestication genes identified are transcription factors, suggesting the importance of expression regulation in the domestication process [3,40,41,42]. This study further confirms that changes in whole-genome expression regulation play a crucial role in the process of divergence between the two subspecies of rice.

## 5. Conclusions

Rice (*O. sativa* L.) is a globally significant cereal crop encompassing two distinct subspecies, *Indica* and *Japonica*, known for their evident phenotypic and population genetic divergence. In this study, we examined 16 quantitative traits across 138 plants under identical conditions, revealing significant disparities between the two subspecies, including key traits like ligule length and plant density. Notably, some traits, such as seeding to the first heading date, exhibited consistency between *tropical japonica* and the *Indica* subspecies, suggesting potential adaptations of *tropical japonica* to higher temperature environments. To investigate the genetic basis of this divergence, we utilized phylogenetic analysis and principal component analysis on resequencing data, affirming the clear divergence between *Indica* and *Japonica*. Furthermore, the neighbor-joining trees based on the expression quantity of effectively expressed genes across various tissues also revealed a consistent pattern with the results based on the resequencing data. We also identified some DEGs between the *Indica* and *Japonica* rice across five tissues. These findings imply that genome-wide expression divergence is influenced by stabilizing selection under non-stressful conditions, mirroring the patterns of sequence variation. In summary, our study confirmed the significant role played by changes in genome-wide expression patterns in driving the divergence between the *Indica* and *Japonica* subspecies of rice. These insights into both phenotypic and genomic distinctions provide valuable contributions to our understanding of rice diversity and adaptation.

## Figures and Tables

**Table 1:** Information of the samples used in the phenotype observation.

No.	Accession No.	Sample Origin	Biological Repetition
TEJ 1 **	680	Zhejiang	8
TEJ 2	IRGC2545	Japan	6
TEJ 10 **	20708	Shanxi	8
TEJ 11 **	20721	Liaoning	6
TEJ 12 **	20876	Jiangsu	7
TEJ 14	21773	Yunnan	4
TEJ 27 *	49357	Guangdong	8
TEJ 28	IRGC55457	Korea	9
TEJ 30	Nipponbare	Japan	8
TRJ 3	IRGC3782	Philippines	2
TRJ 15 **	22128	Guizhou	5
TRJ 22 **	32447	Yunnan	6
TRJ 26	IRGC43675	Indonesia	8
IND 7	IRGC9147	Indonesia	8
IND 13 **	21327	Hubei	7
IND 16 **	22233	Guizhou	7
IND 17 **	24380	Yunnan	6
IND 19	IRGC27748	Thailand	8
IND 24 **	36467	Guangxi	4
IND 25 **	41949	Hainan	5
IND 29 **	60993	Jiangxi	8
Total			138

Note: ** Accessions also used in RNA-seq and resequencing; * Accessions also used in resequencing.

**Table 2:** The phenotypic diversity and intergroup difference for 16 quantitative traits.

Quantitative Traits	CV (Standard Deviation/Mean)			Mean ± Standard Deviation (SD)		
	*Japonica*		*Indica*	*Japonica*		*Indica*
	TEJ	TRJ		TEJ	TRJ	
Days from seeding to first heading date (d)	0.20	0.13	0.26	107.68 ± 21.20 a	122.24 ± 15.66 b	130.36 ± 33.59 b
Ligule length (cm)	0.46	0.41	0.33	1.06 ± 0.49 a	1.81 ± 0.74 b	2.32 ± 0.75 c
Flag leaf length (cm)	0.35	0.29	0.41	21.40 ± 7.53 a	31.71 ± 9.19 b	30.87 ± 12.63 b
Flag leaf width (cm)	0.28	0.22	0.17	1.02 ± 0.28 a	1.55 ± 0.34 b	1.27 ± 0.22 c
Flag leaf attitude (°)	0.63	0.54	0.58	21.89 ± 13.75 a	71.00 ± 38.37 b	26.19 ± 15.17 a
Culm habit (°)	0.65	0.94	0.55	14.57 ± 9.42 a	11.33 ± 10.60 a	21.36 ± 11.78 b
Culm diameter at basal internode (cm)	0.37	0.49	0.46	0.60 ± 0.23 a	0.87 ± 0.42 b	0.92 ± 0.42 b
Culm length (cm)	0.33	0.16	0.22	91.59 ± 30.37 a	133.53 ± 21.26 b	103.82 ± 23.22 c
*Attitude of flowering panicle branches* (°)	6.78	—	5.44	1.09 ± 7.37	0.00 ± 0.00	0.57 ± 3.09
*Attitude of seed setting panicle branches* (°)	3.71	1.92	2.41	2.17 ± 8.07	4.67 ± 8.96	4.50 ± 10.85
*Anther length* (cm)	0.27	0.25	0.27	0.15 ± 0.04	0.16 ± 0.04	0.16 ± 0.04
Peduncle length (cm)	0.87	0.73	1.38	5.48 ± 4.79 a	4.58 ± 3.34 ab	2.76 ± 3.80 b
Panicle length (cm)	0.32	0.25	0.15	17.54 ± 5.55 a	25.92 ± 6.39 b	23.39 ± 3.40 b
Number of spikelets per panicle	0.63	0.44	0.36	67.07 ± 42.20 a	138.38 ± 60.37 b	122.47 ± 44.63 b
Awn length (cm)	2.34	1.34	2.03	0.40 ± 0.92 a	1.12 ± 1.50 b	0.27 ± 0.55 a
*Number of panicles per plant*	0.49	0.47	0.52	5.93 ± 2.93	4.40 ± 2.06	5.53 ± 2.87

Note: 1. The traits highlighted in italics are those with non-significant *p*-values in the ANOVA analysis. 2. In multiple mean comparisons, traits with the same letter label are not significantly different, while traits with different letter labels are significantly different.

**Table 3:** Number of DEGs between *Indica* and *Japonica* rice.

Tissue	EEGs	DEGs		DEGs Total (% of EEGs)
		Up-Regulated	Down-Regulated	
S	23,477	2320	1006	3326 (14.2%)
LH	22,917	1727	919	2646 (11.5%)
PH	26,406	1880	1036	2916 (11.0%)
LM	23,377	2052	1561	3613 (15.5%)
PM	25,260	1699	799	2498 (9.9%)
Mean	24,287	1936	1064	3000 (12.4%)

Note: EEGs: effectively expressed genes; DEGs: differentially expressed genes.

## Data Availability

Not applicable.

## Supplementary Materials

The following supporting information can be downloaded at: https://www.mdpi.com/article/10.3390/genes14101971/s1, Figure S1: Gene ontology (GO) enrichment of the DEGs between *Indica* and *Japonica* rice in PH tissue; Table S1: Information of 95 rice accessions used in this study.
