# Supplementary figures and images for: Differentiation of Morphological Traits and Genome-Wide Expression Patterns between Rice Subspecies Indica and Japonica
Source: Genes (Basel). 2023 Oct 21;14(10):1971. doi: 10.3390/genes14101971 (PMC10606143; doi:10.3390/genes14101971)

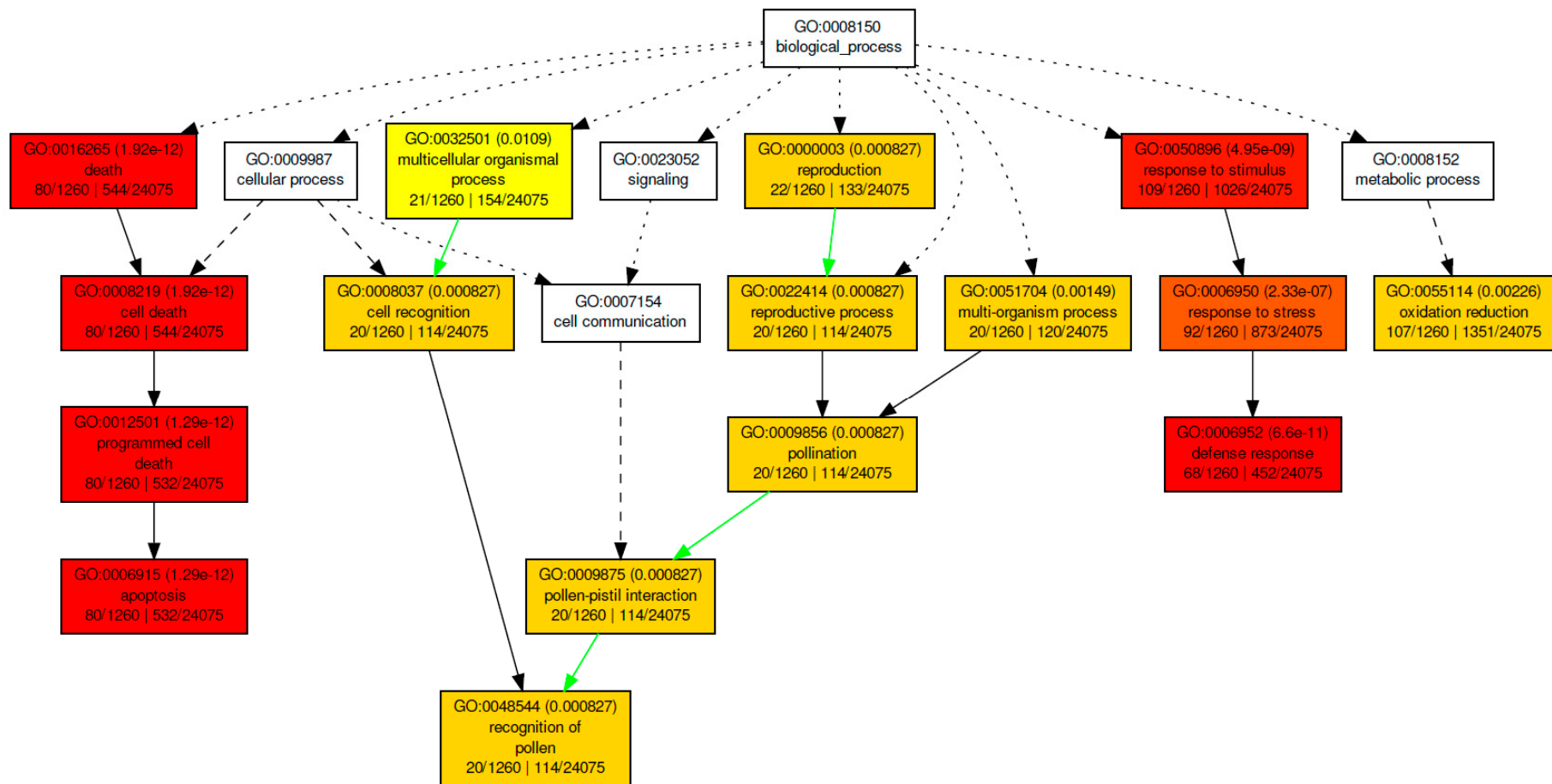

**Figure S1. Gene ontology (GO) enrichment of the DEGs between *Indica* and *Japonica* rice in PH tissue**

Supplement: Supplementary file 1 [file genes-14-01971-s001.zip › Supplemental Figure S1-proofreading.pdf]
